# The Poplar Hospital for Accidents

**Published:** 1907-12-07

**Authors:** 


### THE POPLAR HOSPITAL FOR ACCIDENTS.

### A PROSPEROUS YEAR.

In the *Morning Post* of December 3 the following article appeared:—

There is in London one hospital which declares that it has never been in debt and never will be. If there is another institution of the same kind in the Metropolis it has hitherto hidden its light under a bushel. The one that makes the bold declaration referred to is the Poplar Hospital for Accidents. It does not, however, assert that it will never be in want; it simply says that "if subscriptions fall off, the work will be curtailed and wards closed." There is good reason for hoping that no such dire calamity will befall Poplar, for the hospital is at least fifty-two years old. Its fifty-second annual dinner was held at the Holborn Restaurant last evening, with Sir W. Bull presiding, in the place of Sir Clifton Robinson—obliged to go to the United States after accepting the proposal to take the chair at the banquet. The company included Sir Arthur Douglas, Sir E. Hay-Currie, Mr. Sydney Holland (chairman of the hospital), Mr. Barge (Mayor of Poplar), Mr. W. Crooks, M.P., Mr. A. J. King, M.P., Dr. Theodore Thompson, Mr. O. Magniac, Mr. Edward F. Turner, Mr. W. H. Elwell, Mr. A. Bagnold, Mr. H. Jennings, Mr. W. Baxter, Mr. W. R. L'Amy, Mr. C. Murdock, Mr. Arthur Ross, Mr. W. Nevett, Mr. Seth Taylor, Mr. J. Broadbank, and Mr. Percy Rogers (secretary). The Chairman said that the Poplar Hospital was different from others, inasmuch as it did not indulge in any whining on the subject of money. Mr. Sydney Holland confessed that the hospital had just passed through a very prosperous year. It had been able to spend £3,000 on the Convalescent Home. It had received £3,400 in donations, £2,850 as annual subscriptions, £2,530 as legacies, and £2,000 as interest from investments amounting to £38,500. Of course these figures did not mean that the institution had no need of money. The Secretary announced donations to the amount of £1,600. (Cheers.)

